# Brain volumetric changes and cognitive ageing during the eighth decade of life

**DOI:** 10.1002/hbm.22959

**Published:** 2015-09-07

**Authors:** Stuart J. Ritchie, David Alexander Dickie, Simon R. Cox, Maria del C. Valdes Hernandez, Janie Corley, Natalie A. Royle, Alison Pattie, Benjamin S. Aribisala, Paul Redmond, Susana Muñoz Maniega, Adele M. Taylor, Ruth Sibbett, Alan J. Gow, John M. Starr, Mark E. Bastin, Joanna M. Wardlaw, Ian J. Deary

**Affiliations:** ^1^ Department of Psychology the University of Edinburgh Edinburgh EH8 9JZ United Kingdom; ^2^ Centre for Cognitive Ageing and Cognitive Epidemiology, the University of Edinburgh Edinburgh EH8 9JZ United Kingdom; ^3^ Neuroimaging Sciences, Brain Research Imaging Centre, the University of Edinburgh Edinburgh EH4 2XU United Kingdom; ^4^ Scottish Imaging Network, a Platform for Scientific Excellence (SINAPSE) Collaboration; ^5^ Centre for Clinical Brain Sciences, the University of Edinburgh Edinburgh EH16 4TJ United Kingdom; ^6^ Computer Science Department Faculty of Science, Lagos State University Lagos PMB 001 Nigeria; ^7^ Alzheimer Scotland Dementia Research Centre, the University of Edinburgh Edinburgh EH8 9JZ United Kingdom; ^8^ Department of Psychology Heriot‐Watt University Edinburgh EH14 4AS United Kingdom

**Keywords:** brain volume, structural MRI, age‐related cognitive decline, longitudinal study, white matter hyperintensities

## Abstract

Later‐life changes in brain tissue volumes—decreases in the volume of healthy grey and white matter and increases in the volume of white matter hyperintensities (WMH)—are strong candidates to explain some of the variation in ageing‐related cognitive decline. We assessed fluid intelligence, memory, processing speed, and brain volumes (from structural MRI) at mean age 73 years, and at mean age 76 in a narrow‐age sample of older individuals (*n* = 657 with brain volumetric data at the initial wave, *n* = 465 at follow‐up). We used latent variable modeling to extract error‐free cognitive levels and slopes. Initial levels of cognitive ability were predictive of subsequent brain tissue volume changes. Initial brain volumes were not predictive of subsequent cognitive changes. Brain volume changes, especially increases in WMH, were associated with declines in each of the cognitive abilities. All statistically significant results were modest in size (absolute *r*‐values ranged from 0.114 to 0.334). These results build a comprehensive picture of macrostructural brain volume changes and declines in important cognitive faculties during the eighth decade of life. *Hum Brain Mapp 36:4910–4925, 2015*. © **2015 The Authors. Human Brain Mapping Published by Wiley Periodicals, Inc**

**Abbreviations**
FLAIR: fluid‐attenuated inversion recovery
GM: grey matter
ICV: intracranial volume
WMH: white matter hyperintensities

## INTRODUCTION

Several important cognitive abilities decline with age, even in the absence of dementia or other pathologies [Hedden and Gabrieli, 2004; Tucker‐Drob, 2009]. Given modern demographic shifts [Harper, 2014], cognitive ageing has pressing and increasing economic and social implications for Western societies. Improving our knowledge of the neurobiological underpinnings of ageing‐related cognitive decline is needed to inform future efforts to develop predictive tests and preventative interventions for cognitive ageing [Lindenberger, 2014]. A clear candidate for one of the neuroanatomical bases of cognitive ageing is brain volume. Measures of total brain volume, as well as the volumes of specific tissue types, have consistently been shown to correlate with cognitive ability level throughout adulthood [Arvanitakis et al., in press; Grazioplene et al., 2015; Pietschnig et al., 2014], and changes in brain tissue volumes accompany normal ageing, with healthy tissue volumes declining and white matter hyperintensity (WMH) volumes increasing [Fjell and Walhovd, 2010; Fleischman et al., 2014; Prins and Scheltens, 2015]. In a two‐wave longitudinal study with a large narrow‐age cohort, we investigate the associations between changes in important domains of cognitive ability and in total brain volume, grey matter volume, normal‐appearing white matter volume, and volume of WMH during the eighth decade of life.

In a detailed review of the research on relations between brain anatomy and cognitive abilities during normal, nonpathological ageing, Salthouse [2011] concluded that the overall evidence linking brain volumetric and normal cognitive changes was weak and inconsistent. This is especially true for studies of healthy brain tissue: Only a few longitudinal studies have attempted to estimate either the predictive value of healthy brain tissue volume measured at baseline for subsequent cognitive decline (level‐change correlations; [Cardenas et al., 2011]), or the extent of coupled change in brain volume and cognitive ability (change‐change correlations; e.g. Schmidt et al., 2005). Most relevant for the present analysis, Schmidt et al. [2005] showed in a sample of 329 adults aged approximately 60 years that total brain volume loss across six years was correlated with decline in multiple cognitive skills. Other longitudinal studies have found similar results for loss of tissue in more specific brain regions, such as grey and white matter [e.g. Jokinen et al., 2012; Kramer et al., 2007]. However, some researchers have discovered no change‐change correlations between brain volumes and cognitive abilities: Charlton et al. [2010] found no significant correlation between declines in brain volume and working memory in a two‐year longitudinal study of 84 healthy adults aged 50 to 90 years (see also Du et al., [2006]), which may reflect the shorter duration of follow‐up, age heterogeneity, and smaller sample.

Pathological brain tissue volume, in the form of WMH, has been more extensively studied with reference to cognitive decline. WMH, which are a feature of cerebral small vessel disease, are puncate, focal, or diffuse hyperintense lesions in subcortical regions as seen on T2 and fluid‐attenuated inversion recovery (FLAIR) imaging. Studies have observed differences in several microstructural diffusion parameters in WMH compared to normal‐appearing white matter (lower fractional anisotropy and magnetization transfer ratio, higher mean diffusivity and T1; see Wardlaw et al. [2015] for a detailed discussion of the potential aetiology of WMH and their relation to brain microstructural measurements). In studies including psychometric testing, WMH have been linked to lower cognitive abilities and steeper cognitive decline [Kloppenborg et al., 2014; Prins and Scheltens, 2015]. Whereas some studies have found no clear relation between WMH volume and longitudinal cognitive decline [e.g. Debette et al., 2010], the majority have found level‐change or change‐change correlations between WMH volume and at least one cognitive ability. The small number of studies in the area, however, makes it difficult to draw solid conclusions on the effect sizes involved. For instance, as shown by Kloppenborg et al.'s [2014] meta‐analysis, only four age‐heterogeneous studies have examined change‐change correlations between WMH volume and processing speed, with Fisher's *z* estimates ranging widely, from −0.02 [Schmidt et al., 2005] to −0.42 [Wolfson et al., 2013].

It is unclear whether the inconsistent results in the literature are because of the relatively low statistical power of some previous studies of normal ageing and brain volumetric changes, the possibility that macrostructural neuroanatomical changes are more relevant to dementia‐related cognitive impairments than normal cognitive decline (with affected individuals dropping out of ‘normal’ longitudinal ageing studies), or merely because of the expected statistical variation in results. Many longitudinal studies have used samples with wide age ranges, potentially risking mixing within‐person changes with between‐person differences, masking any true effects [Hofer and Sliwinski, 2001]. In addition, the wide variety of cognitive measures used in different cohorts, even to test the same broad cognitive domains, makes the results less comparable from study to study. Larger‐sample studies, with narrow age ranges and latent‐variable modeling of cognitive abilities (used to capture error‐free variance shared across multiple cognitive tests and thus provide more representative indices of ability than single tests), will provide a clearer picture of the relation of brain tissue changes to cognitive ageing.

Here, in a large, longitudinal, single‐year‐of‐birth cohort study (all participants, who were generally healthy and living independently in the community, were around 73 years old at the first measurement wave, and around 76 at the second) with detailed cognitive and brain MRI data, we addressed two key questions. The first concerned level‐change correlations: do initial levels of brain volume predict differential subsequent change in cognitive abilities, and do initial cognitive ability levels predict subsequent change in brain volume? The second concerned change‐change correlations: are there coupled changes in brain volumes and in cognitive abilities? We tested these hypotheses in a model including only total brain volume (Model A), and then in a more detailed model including grey matter volume, healthy white matter volume, and WMH volume (Model B). In each model, we considered changes in the latent cognitive domains of fluid intelligence, processing speed, and memory. These domains of cognitive ability (as opposed to domains such as crystallized ability, involving measures such as vocabulary) are known to decline appreciably with age [Salthouse, 2004], and thus represent a target for understanding the neurocognitive basis of cognitive ageing.

## METHODS

### Participants

This study reports analysis of data collected from members of the Lothian Birth Cohort 1936 (LBC1936; [Deary et al., 2012, 2007]), a longitudinal study of ageing in the Edinburgh and Lothians area of Scotland, UK. Written informed consent was obtained from all participants, and the overall study was approved by the Lothian Research Ethics Committee (LREC/2003/2/29) and the Multi‐Centre Research Ethics Committee for Scotland (MREC/01/0/56). At Wave 1, 1,091 participants had cognitive and other data measured at a mean age of 69.53 years (SD = 0.83); Wave 1 data are not included in the present study, because neuroimaging data were available at only Waves 2 and 3 (see below). At Wave 2 of testing, 866 participants (337 female) attended at a mean age of 72.49 years (SD = 0.71), when the same cognitive tests were repeated. At Wave 3, these tests were repeated again; 697 participants (337 female) attended at a mean age of 76.25 years (SD = 0.68).

Seven hundred and thirty one participants (343 female) attended brain MRI scanning at Wave 2 (mean age = 72.68 years, SD = 0.72; mean time lag between cognitive testing and MRI scanning at Wave 2 = 65 days, SD = 40). At Wave 3, 488 participants (228 female) attended for brain MRI scans, at a mean age of 76.38 years (SD = 0.65; mean time lag between cognitive testing and MRI scanning at Wave 3 = 40 days, SD = 32). There were a variety of reasons for nonattendance at the second scanning wave; our records show that 38 participants died at some point in the intervening three years. Twenty‐nine subjects with repeated scans were excluded due to incomplete MRI and/or motion artefacts that were too severe to be overcome with motion correction algorithms. Four hundred and fifty nine subjects had full brain MRI data at both waves. See Table 1 for sample size details for all measures.

**Table 1:** Descriptive statistics for each variable measured longitudinally, with factor loadings from the final model for each cognitive variable

Variable type	Variable	*n*		Mean (SD)		Factor loading (*λ*)
		Age 73	Age 76	Age 73	Age 76	
Brain volumes	Total brain volume (cm^3^)	657	465	990.32 (89.40)	975.49 (90.49)	–
	Grey matter volume (cm^3^)	657	461	472.43 (44.68)	465.67 (43.61)	–
	NAWM volume (cm^3^)	657	461	476.89 (50.55)	464.25 (53.10)	–
	WMH volume (cm^3^)	656	464	12.23 (12.18)	15.85 (14.57)	–
Fluid intelligence	Matrix reasoning	863	689	13.17 (4.96)	13.04 (4.91)	0.651
	Block design	864	691	33.64 (10.08)	32.18 (9.95)	0.672
	Digit span backward	866	695	7.81 (2.29)	7.77 (2.37)	0.582
	Letter‐number sequencing	863	687	10.91 (3.08)	10.48 (2.99)	0.649
Memory	Logical memory	864	688	74.23 (17.90)	74.58 (19.20)	0.629
	Verbal paired associates	843	663	27.18 (9.46)	26.41 (9.56)	0.572
	Spatial span	861	690	14.70 (2.76)	14.62 (2.73)	0.532
Speed	Digit‐symbol substitution	862	687	56.40 (12.31)	53.81 (12.93)	0.521
	Symbol search	862	685	24.61 (6.18)	24.60 (6.46)	0.427
	Simple reaction time (s)	865	688	0.275 (0.052)	0.283 (0.052)	0.509
	Choice reaction time (s)	865	685	0.649 (0.089)	0.678 (0.103)	0.795
	Inspection time	838	654	111.22 (11.79)	110.17 (12.53)	0.523

Abbreviations: NAWM, Normal‐appearing white matter; WMH, White matter hyperintensity. Factor loadings are from Model A (including only total brain volume), but were very similar in Model B. All factor loadings significant at *P* < .001. The invariance assumption meant that factor loadings were identical at both waves. Digit‐symbol substitution and symbol search also (cross‐) loaded on Fluid Intelligence (loadings = 0.316 and 0.384, respectively).

At entry to the study, none of the participants had a diagnosis of dementia. Below, we describe a sensitivity analysis using a dementia screening instrument and a set of dementia ascertainment methods to exclude those participants who may subsequently have developed dementia after the beginning of the study.

### Measures

#### Cognitive testing

Three domains of cognitive function were tested, and common factors were extracted using latent variable modeling. The first, fluid intelligence, was made up of the Matrix Reasoning, Block Design, Digit Span Backward and Letter‐Number Sequencing subtests from the Wechsler Adult Intelligence Scale, 3^rd^ UK Edition (WAIS‐III^UK^; [Wechsler, 1998a]). These tests assess reasoning and working memory. The second domain, Memory, included the Logical Memory (immediate and delayed), Verbal Paired Associates, and Spatial Span (forward and backward) tests from the Wechsler Memory Scale, 3^rd^ UK Edition (WMS‐III^UK^; [Wechsler, 1998b]). These tests examine episodic, verbal, and spatial memory. The third domain was Speed, which included two paper‐and‐pencil tests (Digit‐Symbol Substitution and Symbol Search from the WAIS‐III^UK^), two tests using a dedicated reaction time instrument (Simple and Four‐Choice Reaction Time; [Deary et al., 2001]), and one computer‐based psychophysical test of visual processing efficiency (Inspection Time; [Deary et al., 2004]). In the model, latent variables were derived for three cognitive domains (and their fit to the data tested; see ‘Statistical Analysis’ subsection, below). To improve model fit, we included cross‐loadings of the two pencil‐and‐paper tests of speed—Digit‐Symbol Substitution and Symbol Search—on the Fluid Intelligence factor as well as the Speed factor. In addition, the cohort members were all tested on the Mini‐Mental State Examination (MMSE; [Folstein et al., 1975]), a test often used to screen for possible dementia, and used here in a sensitivity analysis.

#### Brain MRI acquisition and processing

Full brain MRI acquisition parameters have been described previously [Wardlaw et al., 2011]. Briefly, all subjects had brain MRI using the same 1.5 Tesla GE Signa Horizon HDx clinical scanner (General Electric, Milwaukee, WI, USA) at Waves 2 and 3 (73 and 76 years); mean time between scanning was 3.7 years (SD = 0.26, range = 2.2–4.8). The scanning protocol was the same at 73 and 76 years, and the scanner underwent a careful longitudinal quality assurance program to minimize changes in its performance over time. The acquired 3D T1‐weighted volume, and T2‐, T2*‐, and fluid attenuated inversion recovery (FLAIR)‐weighted axial scans were all co‐registered [Jenkinson and Smith, 2001] at 1 × 1 × 2 mm^3^ to allow easier identification of different brain structures.

We measured intracranial volume (ICV), whole brain, grey matter (GM), normal‐appearing white matter, and WMH volumes using a validated multispectral image processing method that uses intensities from T1‐, T2‐, T2*‐, and FLAIR‐weighted MRI sequences for segmentation. Briefly, all sequences were co‐registered and tissue volumes quantified using Gaussian mixture modeling cluster analysis [Valdés Hernández et al., 2010; Wardlaw et al., 2011]. According to Standards for Reporting Vascular changes on nEuroimaging (STRIVE), we explicitly defined WMH as punctate, focal, or diffuse lesions in all subcortical regions [Wardlaw et al., 2013]. We did not treat periventricular and deep WMH separately, as has been done in some previous studies, because these have been shown to be highly correlated [e.g. DeCarli et al., 2005 found the correlation to be around *r* = *0*.9] and their division may be arbitrary: for example, periventricular WMH are often contiguous with superior deep WMH.

Blinded to all clinical and cognitive details, we manually checked all segmented images for accuracy, corrected errors, and excluded imaging‐detected infarcts from WMH volumes. Infarcts are hyperintense on FLAIR, and may therefore be picked up erroneously by most WMH algorithms; they thus have to be removed manually. Imaging‐detected infarcts included historical and/or silent stroke lesions that occurred in approximately 13% of participants (58 of the 459 participants with repeated MRI data); these were excluded from all tissue volumes. This incidence is approximately equal to the average incidence of infarcts in other large ageing cohorts [Schmidt et al., 2005; van Dijk et al., 2008]; we did not exclude these participants to ensure that our sample was as representative as possible of the wider population of older adults. Infarcts were identified by their cortical wedge‐shaped distribution in a typical arterial territory or, if subcortical, by their size larger than 2 cm indicating a striatocapsular aetiology. They were removed from WMH masks using the 3D mask‐editing software Multi‐Image Analysis GUI (MANGO; http://ric.uthscsa.edu/mango/). Editing was guided by instructions from a consultant neuroradiologist (author J.M.W.) [Wang et al., 2012; Wardlaw et al., 2013].

**Figure 1:**
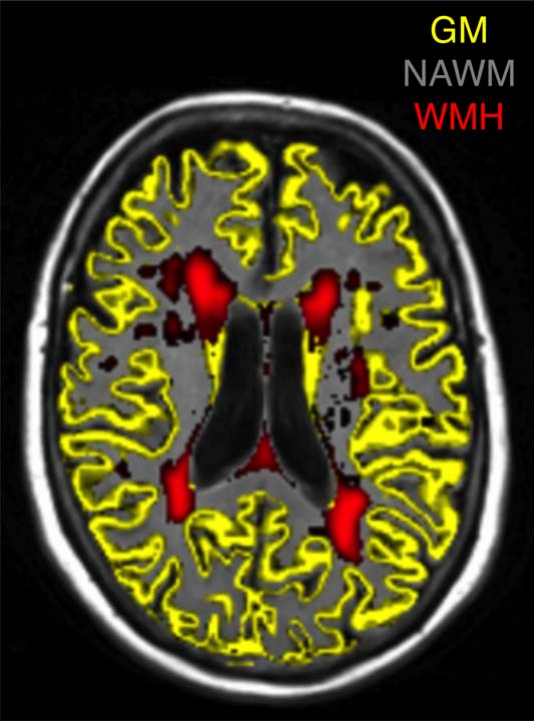
Use of multisequence (T1‐weighted and FLAIR‐weighted) brain magnetic resonance imaging (MRI) to produce tissue‐segmented images in one randomly‐selected participant from the present study. GM, grey matter; NAWM, normal‐appearing white matter; WMH, white matter hyperintensities.

See Figure 1 for an example brain image from the processing pipeline described above. All results below are calculated for raw tissue volumes; however, using volumes at each age as a proportion of ICV (to correct for differences in head size and in atrophy before the beginning of the study) did not substantially alter the findings reported here. In addition, log‐transforming the positively‐skewed WMH distributions had little effect on our results.

### Statistical Analysis

**Figure 2:**
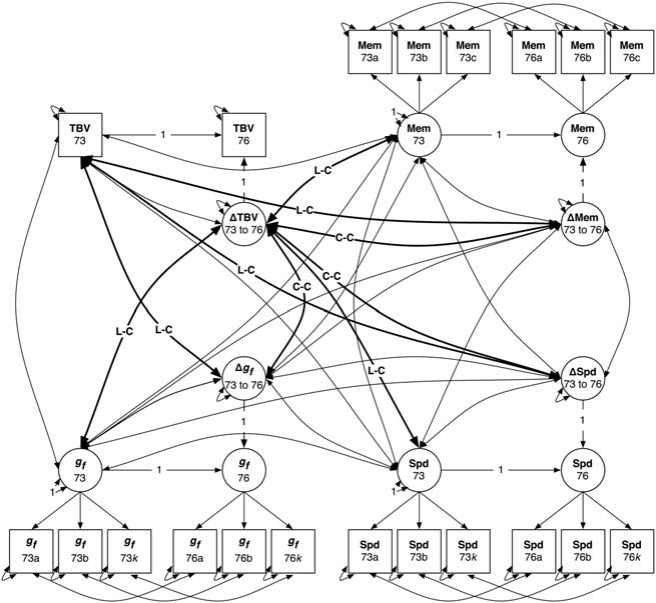
Latent difference score model diagram for Model A, including total brain volume (TBV). Measured (manifest) variables are shown as squares, and latent variables as circles. Each manifest variable is measured at both waves (mean age 73 and 76, respectively); for each cognitive domain, fluid intelligence (*g*_f_), speed (Spd), and memory (Mem), the manifest variables a‐*k* indicate a latent variable (for simplicity, only three indicators are shown; see Materials and Methods for all cognitive tests used). Latent change variables (ΔTBV, Δ*g*_f_, ΔMem, and ΔSpd) are calculated within the model. The main paths of interest are the relations between initial TBV level at 73 and subsequent change in cognitive abilities, and vice‐versa (bold paths labeled L‐C for level‐change), and between change in brain volume and change in cognitive abilities (bold paths marked C‐C for change‐change).

Structural equation modeling was used to estimate the associations between changes in brain tissue volume and changes in cognitive abilities from age 73 to 76. We used a latent difference score model [McArdle, 2009], which is appropriate for use with two‐wave longitudinal data. For the cognitive tests, a latent variable was extracted at each wave to capture the shared variance among the tests and indicate ability in each of the three domains (Fluid Intelligence, Memory, and Speed). A change score (Δ) representing people's relative decline for each factor from age 73 to age 76 was then estimated. This variable represents the part of the age 76 latent variable that is not explained by the age 73 latent variable. Because change, for the cognitive factors, was estimated from two latent variables, it was free of measurement error specific to the individual cognitive tests. We could not use latent variable modeling for brain volume change, where the change score was estimated from two manifest variables (i.e., there was a single volumetric measurement at each wave). The model including total brain volume (Model A) is illustrated in Figure 2. A second model (Model B) was also estimated using separate grey matter, white matter, and WMH changes in place of the single total brain volume change. This model assessed changes in six variables, as opposed to the four shown in Figure 2. The models allow the testing of level‐change correlations (does the initial level of one variable predict the subsequent degree of change in another?) and change‐change correlations (do the two variables change together?).

On the basis of a previous analysis [Ritchie et al., 2015], we modeled the cognitive factors as having strong measurement invariance (thus measuring the same construct across the two waves; [Widaman et al., 2010]). Specifically, this involved fixing the factor loadings and the intercepts of each of the tests to be equal across waves. To reduce the influence of confounding factors, we controlled all of the variables for sex and age (the latter measured in days at the time of cognitive testing or of MRI scanning, as appropriate), before including them in the model. For the analyses of sex differences, the variables were only controlled for age. All modeling was performed using Mplus v7.3 [Muthén and Muthén, 1998‐2014], using full‐information maximum likelihood estimation to take into account all of the data. An analysis including only participants who had contributed data at both waves (i.e. a ‘complete‐cases’ analysis) did not produce results that were substantially different to those reported below (in most cases producing slight increases in the effect sizes).

To test whether the sizes of the level‐change or change‐change relations differed significantly from one cognitive domain to another, and from one brain tissue type to another, we produced new models where these relations were constrained to equality, and tested their fit against the original, unconstrained model (using a chi‐squared test). We performed this test only where one or both of the paths were statistically significant. Finally, as an exploratory analysis, we tested for sex differences in the sizes of any (overall significant) correlations by testing whether sex interacted with the predictor variable (e.g. age 73 cognitive ability) to predict the outcome (e.g. total brain volume change).

## RESULTS

Descriptive statistics, and the loading of each cognitive test on its respective latent cognitive factor, are provided in Table 1, and the brain volumes at each wave of the study are shown split by sex in Table 2. Cross‐wave changes are analyzed below; the loadings of the cognitive abilities ranged from 0.316 to 0.795 (mean loading = 0.554).

**Table 2:** Mean total brain volumes and volumes of individual brain tissues at each wave of the study, grouped by sex

Brain tissue type	Males		Females	
	Age 73 Mean (SD)	Age 76 Mean (SD)	Age 73 Mean (SD)	Age 76 Mean (SD)
Total brain volume (cm^3^)	1038.59 (77.68)	1022.97 (80.10)	934.62 (66.85)	919.53 (67.10)
Grey matter volume (cm^3^)	496.57 (38.61)	487.25 (39.32)	444.57 (33.66)	440.54 (33.86)
NAWM volume (cm^3^)	501.13 (44.85)	490.28 (47.61)	448.91 (41.53)	433.94 (41.96)
WMH volume (cm^3^)	12.00 (11.55)	16.05 (14.50)	12.49 (12.89)	15.61 (14.67)

Abbreviations: NAWM, Normal‐appearing white matter; WMH, White matter hyperintensity.

A correlation matrix showing the longitudinal stability of each of the variables as well as their cross‐sectional relations is shown in Table 3. All of the brain volume measures and cognitive factors were highly stable across the waves (all *r*‐values > 0.867). As has already been reported in this dataset [Royle et al., 2013; Valdés Hernández et al., 2013], each of the brain volume measures was significantly correlated with each of the cognitive abilities at age 73: total, grey, and white matter volumes had positive correlations with cognitive ability (mean *r* = 0.278, *P*‐values < 0.001), whereas WMH volume correlated negatively with all three cognitive factors (mean *r* = −0.186, all *P*‐values < 0.002). This pattern was replicated in the new data from age 76: positive correlations with cognitive ability were found for total, grey, and white matter volumes (mean *r* = 0.299, *P*‐values < 0.001), and negative correlations were found for WMH volume (mean *r* = −0.207, all *P*‐values < 0.002; Table 3).

**Table 3:** Correlation matrix for the brain variables and latent cognitive variables at age 73 (below the diagonal) and age 76 (above the diagonal), with values on the diagonal showing the within‐variable correlation from age 73 to age 76

Variable	Total brain volume	Grey matter volume	NAWM volume	WMH volume	Fluid intelligence	Memory	Speed
Total brain volume	0.966***	0.869***	0.835***	0.141**	0.323***	0.252***	0.267***
Grey matter volume	0.877***	0.925***	0.629***	−0.115**	0.339***	0.288***	0.271***
NAWM volume	0.863***	0.663***	0.949***	−0.239***	0.334***	0.269***	0.350***
WMH volume	0.099*	−0.086*	−0.264***	0.970***	−0.179***	−0.174**	−0.267***
Fluid intelligence	0.301***	0.314***	0.300***	−0.152**	0.969***	0.910***	0.707***
Memory	0.225***	0.257***	0.230***	−0.172***	0.860***	0.896***	0.675***
Speed	0.273***	0.270***	0.336***	−0.233***	0.606***	0.588***	0.867***

*P < 0.05, **P < 0.01, ***P < 0.001.

All correlations controlled for sex and age (in days at testing or at MRI scanning). Correlations between total brain volume and other brain volumes were calculated outside of the structural equation models.

Abbreviations: NAWM, Normal‐appearing white matter; WMH, White matter hyperintensity.

The 169 participants (19.5% of the original 866) who did not return for the age‐76 cognitive testing had significantly lower scores on fluid intelligence (*t*(252.100) = 5.700, *P* < 0.001), Memory (*t*(241.085) = 5.644, *P* < 0.001), and Speed (*t*(228.537) = 4.734, *P* < 0.001). The 250 participants (34.2% of 731) who were scanned at age 73 but did not return for a second scan had lower grey matter volume (*t*(379.374) = 2.760, *P* = 0.006), but did not differ significantly in white matter volume (*t*(352.756) = 0.682, *P* = 0.495), white matter hyperintensity volume (*t*(316.226) = 0.899, *p* = 0.369), or total brain volume (*t*(379.199) = 1.661, *P* = 0.100).

### Changes in Brain Volumes and Cognitive Abilities

Using only the paths shown in Figure 2, the structural equation models fit the data acceptably, with Root Mean Square Error of Approximation (RMSEA) values close to 0.06 and Comparative Fit and Tucker‐Lewis Indices (CFI and TLI) close to 0.95 [Hu and Bentler, 1999]: Model A (total brain volume only; Figure 2) *χ* ^2^(276) = 917.990, *p* < .0001, RMSEA = 0.052, CFI = 0.942, TLI = 0.931; Model B (grey matter, white matter, and hyperintensity volumes separately) *χ* ^2^(348) = 1011.757, *P* < 0.001, RMSEA = 0.047, CFI = 0.950, TLI = 0.937.

**Figure 3:**
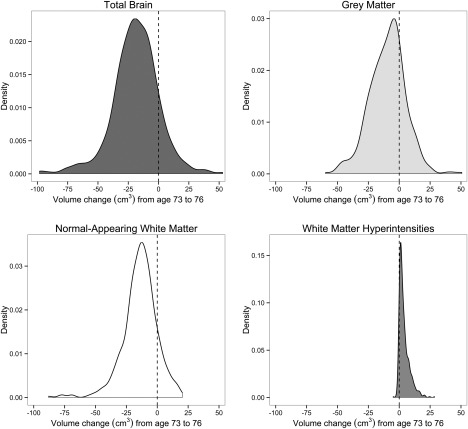
Density plots showing longitudinal change (from age 73 to age 76) in total brain volume (upper left), grey matter volume (upper right), normal‐appearing white matter volume (lower left), and white matter hyperintensity volume (lower right). The dashed line on each plot's *x*‐axis indicates zero change in volume.

Within the models, we calculated the mean decline in each of the brain variables across the two waves of the study. All brain volumetric measurements exhibited significant change between ages 73 and 76 (Table 4): total brain volume, grey matter volume, and white matter volumes declined (by 0.64% to 1.01% of their original volumes per year on average), whereas WMH volume increased across time (adding 11.04% of the original WMH volume per year on average). Density plots illustrating the distributions of longitudinal change in each of the volumetric measurements are shown in Figure 3.

**Table 4:** Changes in brain volumes, estimated from the structural equation model, on different metrics

Brain tissue type	Mean volume change [95%CI]					
	cm^3^ across study	cm^3^ per year	SDs per year	% change per year	*z*	*p*	
Total brain	−19.12 [−20.88, −17.35]	−6.37 [−6.96, −5.78]	−0.071 [−0.079, −0.063]	−0.64% [−0.70%, −0.58%]	−21.22	<0.001	
Grey matter	−9.28 [−10.62, −7.95]	−3.09 [−3.53, −2.65]	−0.069 [−0.080, −0.059]	−0.65% [−0.75%, −0.56%]	−13.66	<0.001	
NAWM	−14.46 [−15.75, −13.18]	−4.82 [−5.25, −4.39]	−0.096 [−0.105, −0.086]	−1.01% [−1.10%, −0.92%]	−22.07	<0.001	
WMH	+4.06 [+3.68, +4.44]	+1.35 [+1.23, +1.48]	+0.111 [+0.110, +0.123]	+11.04% [+10.06%, +12.10%]	+21.09	<0.001	

Abbreviations: NAWM = normal‐appearing white matter; WMH = white matter hyperintensity; 95%CI = 95% confidence interval; SDs = standard deviations.

Male cohort members had steeper decline in their total brain volume (0.69% of the original volume per year versus 0.57% per year in female cohort members; *t*(836.79) = 3.56, *P* < 0.001, *d* = 0.25). This was driven by grey matter loss, of which male cohort members also experienced a greater rate than females (losing 12.43% per year compared to 5.74% in females; *t*(850.63) = 6.24, *P* < 0.001, *d* = 0.43) There were no significant sex differences in the rate of change in normal‐appearing white matter (0.91% loss per year in males versus 1.13% in females; *t*(795.23) = 1.43, *P* = 0.15), or in WMH change (11.61% increase in males versus 10.54% in females; *t*(862.81) = 0.92, *P* = 0.36).

Each of the cognitive factors showed significant decline across the two waves of the study from mean age 73 to 76 (Fluid Intelligence: 0.074 SDs/year, *z* = −8.964, *P* < 0.001; Memory: 0.058 SDs/year, *z* = −4.814, *P* < 0.001; Speed: 0.147 SDs/year; *z* = −12.818, *P* < 0.001). As would be expected from studies showing a general factor of cognitive change [e.g. Tucker‐Drob, 2011], the cognitive abilities declined together, with relatively strong correlations between their changes (mean *r* = 0.634; all cognitive change values above are from Model A with only total brain volume, but were very similar in Model B, with the three different brain tissue types).

### Associations Between Levels and Changes Within Brain Volumes

In Model A, those with higher levels of total brain volume at age 73 tended to have greater decline in total brain volume to age 76 (*r* = −0.121, *P* = 0.007; possibly due to the ‘law of initial value’; [Wilder, 1957]). Table 5 shows the level‐change and change‐change correlations among the volumes of the three brain tissue types measured in Model B. As for total volume in Model A, higher initial grey matter volumes predicted more decline in grey matter (*r* = −0.182, *P* < 0.001), but not either white matter or WMH (*P*‐values > 0.175). Initial normal‐appearing white matter volume was predictive of WMH change, but not grey or white matter change (*P*‐values > 0.430). WMH level at age 73 was predictive of changes in all three tissue types (mean *r* = 0.334, all *P*‐values < 0.012), but it most strongly predicted WMH change: individuals with more hyperintensities at age 73 tended to have greater development of hyperintensities to age 76 [see also Dickie et al., under review]. As is also shown in Table 5, WMH volume change was also significantly correlated with change in both grey and normal‐appearing white matter (mean *r* = 0.262, *P*‐values < 0.003). Change in normal white matter volume was not correlated with change in grey matter volume (*P* = 0.194).

**Table 5:** Level‐change and change‐change correlations amongst grey matter, white matter, and white matter hyperintensity volumes (from Model B)

	Grey matter volume change	NAWM volume change	WMH volume change
Grey matter volume level	−0.182***	−0.062	0.009
NAWM volume level	0.036	−0.010	−0.102*
WMH volume level	−0.121*	−0.285***	0.596***
NAWM volume change	0.060	–	–
WMH volume change	−0.140**	−0.384***	–

*P < .05, **P < .01, ***P < .001.

NAWM, normal‐appearing white matter; WMH, white matter hyperintensity. All brain volumes controlled for sex and for age in days at MRI scanning.

### Level‐Change Associations Between Brain Volumes and Cognitive Abilities

**Figure 4:**
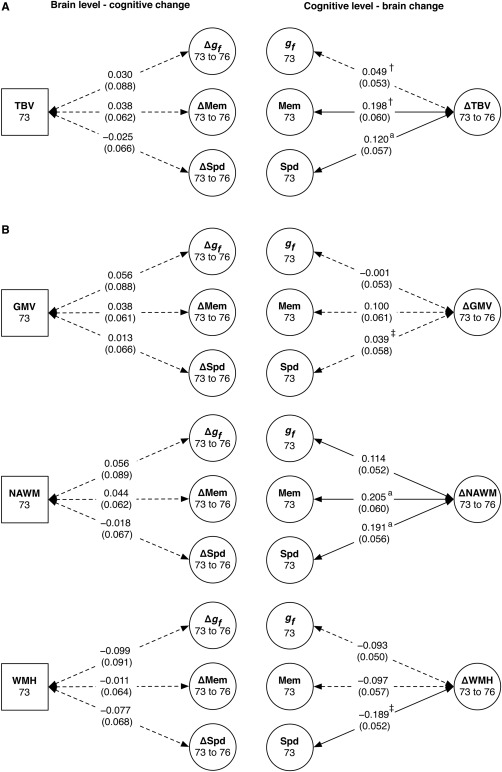
Level‐change associations between brain volumes and cognitive abilities for (**A**) Model A, including total brain volume and (**B**) Model B, including grey matter, white matter, and white matter hyperintensity volumes. The left column shows associations between age‐73 total, grey matter, normal‐appearing white matter, and white matter hyperintensity volumes (TBV, GMV, NAWM, and WMH, respectively) and change across the subsequent three years in fluid intelligence (Δ*g*_f_), memory (ΔMem), and speed (ΔSpd). The right column shows associations between age‐73 cognitive abilities and change in total, grey matter, white matter, and WMH volumes. Values are standardized path estimates, with standard errors in parentheses. Dashed lines indicate paths that were not statistically significant at *P* < .05. Where the symbols † and ‡ appear, the other path with the same symbol had a significantly different effect size. Where the superscript ^a^ appears, the path was significantly stronger in male cohort members.

We then tested our main hypotheses, on brain‐cognitive ability relations. The level‐change associations between brain volumes and cognitive abilities are shown in Figure 4, for Models A (total brain volume only; upper section) and B (grey matter, white matter, and hyperintensity volumes separately; lower section). All associations between brain volume levels at age 73 and cognitive change between 73 and 76 (Figure 4, left column) were non‐significant: for total brain volume, all *P*‐values were > 0.536; for grey matter volume, all *P*‐values > 0.526; for white matter volume, all *P*‐values > 0.473; and for WMH volume, all *P*‐values > 0.262. That is, brain volumetric measurements taken at age 73 did not predict the degree of cognitive decline across the next three years.

However, there were significant results among the converse associations: the level of some cognitive abilities at age 73 significantly predicted changes in brain volumes from age 73 to age 76 (Figure 4, right column). In Model A, higher age‐73 levels of Memory and Speed (mean *r* = 0.159, *P*‐values < 0.036), but not Fluid Intelligence (*P* = 0.358), predicted less decline in total brain volume, and the relation with Memory was significantly stronger than that for Fluid Intelligence (*χ* ^2^(1) = 8.133, *P* = 0.004). Males had a stronger correlation between their age 73 Speed and subsequent decline in total brain volume than did females (standardized *β* for interaction = 0.155, *P* = 0.021), but this was not the case for Memory (*β* = 0.080*, P* = 0.229). In Model B, higher levels in all three cognitive domains predicted less decline in white matter volume (mean *r* = 0.170, *P*‐values < 0.031), and males had significantly stronger correlations than females for Memory (*β* = 0.139*, P* = 0.037) and Speed (*β* = 0.188*, P* = 0.005). Higher levels of Speed, but not the other cognitive domains, predicted less WMH growth (*r* = 0.189, *P* < 0.001). There were no significant associations between cognitive scores at age 73 and grey matter changes between age 73 and age 76 (all *P*‐values > 0.104). Baseline Speed more strongly predicted subsequent change in WMH than change in grey matter (*χ* ^2^(1) = 5.088, *P* = 0.024).

### Correlated Changes in Brain Volumes and Cognitive Abilities Between Ages 73 and 76

**Figure 5:**
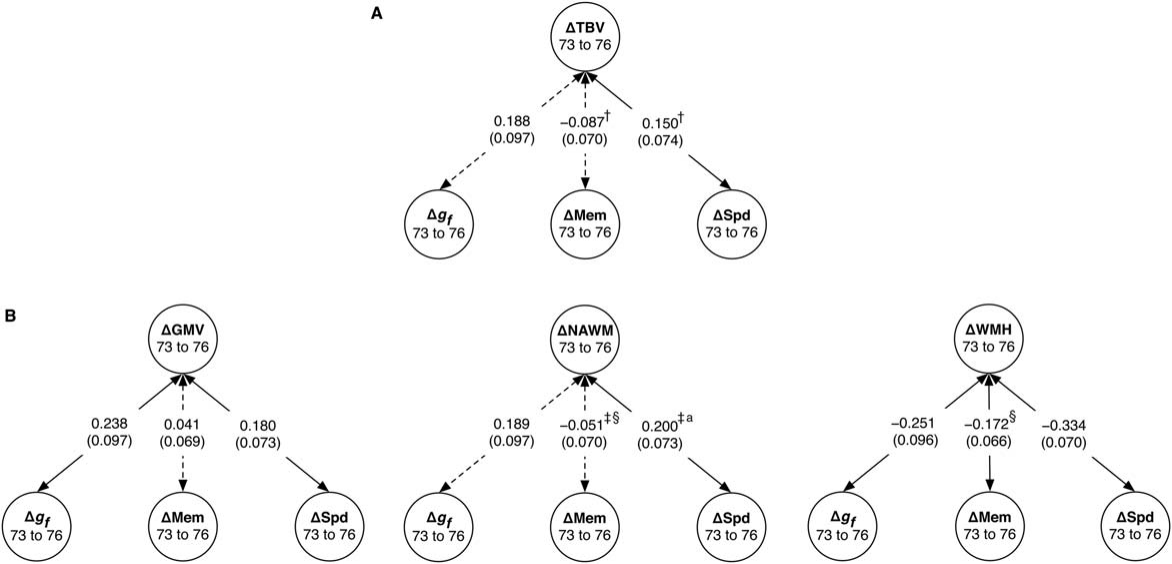
Change‐change associations between brain volumes and cognitive abilities from age 73 to age 76. Associations are shown for (**A**) Model A, including change in total brain volume (ΔTBV) and (**B**) Model B, including change in grey matter, normal‐appearing white matter, and white matter hyperintensity volumes (ΔGMV, ΔNAWM, and ΔWMH, respectively). Associations are shown with change in fluid intelligence (Δ*g*_f_), memory (ΔMem), and speed (ΔSpd). Values are standardized path estimates, with standard errors in parentheses. Dashed lines indicate paths that were not statistically significant at *P* < 0.05. Where the symbols †, ‡, and § appear, the other path with the same symbol had a significantly different effect size. Where the superscript ^a^ appears, the path was significantly stronger in male cohort members.

We then tested the change‐change correlations between brain and cognitive ability. The results of these tests are shown in Figure 5. The model including total brain volume (Model A; Figure 5, upper section) showed that greater loss of total brain volume was significantly, though marginally, correlated with more decline in Speed (*r* = 0.150, *P* = 0.043), but not with Fluid Intelligence (*r* = 0.188, *P* = 0.052; though the effect sizes were similar) or Memory (*r* = −0.087, *P* = 0.211). The size of the change‐change correlation with Speed was significantly larger than that with Memory (*χ* ^2^(1) = 6.844, *P* = 0.009). There were no significant sex differences in Model A.

There were several significant correlations in Model B, which examined different brain tissues separately (Figure 5, lower section). These were between loss of grey matter volume and decline in Fluid Intelligence (*r* = 0.238, *P* = 0.015) and Speed (*r* = 0.180, *P* = 0.014), but not with decline in Memory (*r* = 0.041, *P* = 0.556). White matter change was correlated only with change in Speed (*r* = 0.200, *P* = 0.006), and not with Fluid Intelligence (*r* = 0.189, *P* = 0.051) or Memory (*r* = −0.054, *P* = 0.439). As with total brain volume, the correlation with Speed was significantly stronger than that with Memory (*χ* ^2^(1) = 7.878, *P* = 0.005), and again, Speed and Fluid Intelligence exhibited similar effect sizes. Increases in WMH volume from 73 to 76 were significantly correlated with declining performance in all three cognitive domains: Fluid Intelligence (*r* = −0.251, *P* = 0.009), Memory (*r* = −0.172, *P* = 0.010), and Speed (*r* = −0.334, *P* < 0.001). The change‐change correlation between WMH volume and Memory was significantly larger than that between normal‐appearing white matter volume and Memory (*χ* ^2^(1) = 8.509, *P* = 0.004), but the other correlations (e.g. with Speed and with Memory) did not differ significantly from one another.

### Analyses Correcting for Multiple Comparisons

Due to the multivariate nature of the models, many hypotheses were tested in each. For this reason, we applied a False Discovery Rate correction for multiple comparisons [Benjamini and Hochberg, 1995] across all the level‐level, level‐change, and change‐change correlation *P*‐values in each of the two models. In Model A, twelve of the fourteen previously‐significant correlations remained significant: the correlations between change in total brain volume and the level and change in speed were both reduced to non‐significance (original *P*‐values = 0.035 and 0.043, respectively; corrected *P*‐values = 0.075 and 0.086, respectively). In Model B, only one previously‐significant correlation became non‐significant after correction, namely the correlation between fluid intelligence level and change in white matter (original *P*‐value = 0.030; corrected *P*‐value = 0.055); the other thirty‐five previously‐significant correlations remained so.

### Analyses Excluding Participants with Low MMSE Scores and Dementia Diagnoses

There is a possibility that longitudinal relations between brain and cognitive ability are different in individuals in the prodromal or early phase of neuropathological conditions such as dementia. For this reason, we ran two sensitivity analyses. In the first (‘MMSE‐exclusion’), we excluded 16 individuals who scored below 24 on the MMSE at either or both of the testing waves. In the second (‘diagnosis‐exclusion’), we excluded 25 individuals who we ascertained had been given a formal diagnosis of dementia (by inspection of death certificates where relevant, participants' health status on the Scottish Morbidity Records, inpatient psychiatric records, or as a part of clinical reviews conducted as part of the LBC1936 study).

For the level‐change results, the MMSE‐exclusion sensitivity analysis reduced the predictive correlation in Model A between baseline Speed and subsequent total brain volume change to non‐significance (*r* = 0.097, *P* = 0.229). The same was true, to a lesser degree, in the diagnosis‐exclusion analysis (*r* = 0.110, *P* = 0.052). The correlation between baseline Memory and brain volume change was attenuated, but remained significant in both sensitivity analyses (*r* = 0.136, *P* = 0.030 for MMSE‐exclusion; *r* = 0.187, *P* = 0.002 for diagnosis‐exclusion). In Model B, the correlation between baseline Fluid Intelligence and white matter change was reduced to non‐significance in the MMSE‐exclusion sensitivity analysis (r = 0.072, *P* = 0.196), but not the diagnosis‐exclusion analysis (*r* = 0.111, *P* = 0.032); the other three previously‐significant correlations remained significant, though with somewhat smaller effect sizes.

For the change‐change correlations, in Model A there were no longer any significant correlations after the MMSE‐exclusion analysis: the correlation with Speed was no longer significant (*r* = 0.097, *P* = 0.229). However, this correlation was still significant in the dementia‐exclusion analysis (*r* = 0.154, *P* = 0.042). In Model B, the only previously‐significant change‐change correlation that was reduced to non‐significance was that between white matter change and Speed change in the MMSE‐exclusion analysis (*r* = 0.092, *P* = 0.249). All other previously‐significant change‐change correlations remained significant in both sensitivity analyses, with only small differences in their effect sizes.

## DISCUSSION

Two well‐replicated findings—that brain tissue volumes correlate with cognitive ability levels, and that both cognitive ability and brain volume change during normal ageing—suggest that there could be longitudinal links between structural brain volumes and cognitive test scores during later life. We examined these links in a large, narrow‐age cohort of older adults with repeated neuroimaging and cognitive testing data at mean ages 73 and 76. We found evidence for level‐change and change‐change correlations. Levels of Memory and Speed assessed at baseline (age 73) were predictive of subsequent loss of healthy brain tissue, and growth of WMH. There were also modest, but significant, coupled changes between age 73 and age 76 in brain volumes and in all three cognitive domains assessed. Ageing‐related atrophy of healthy tissue, and progression of WMH, thus advanced alongside the loss of important cognitive abilities. This occurred in the absence of any behavioral indications or medical diagnoses of dementia, indicating that these results are relevant to the normal, nonpathological ageing process (although after excluding participants with indications of dementia, effect sizes were somewhat attenuated). Whereas our correlational analysis does not allow us to draw causal conclusions about brain‐cognitive relations, it sheds light on the particular cognitive abilities that decline in concert with changes in brain macrostructure, and the strengths of the correlations between the changes.

Participants with higher cognitive abilities at age 73 showed less brain volume loss and less lesion growth over the next three years. That is, cognitive tests (of Memory and Speed) were a predictor of less subsequent change in brain volume. It is possible that this is due to the established relations of cognitive ability with health behaviors: for instance, individuals with better cognitive ability tend to smoke less and exercise more (Gottfredson, 2004), and these lifestyle factors may impact brain structure (e.g. [Almeida et al., 2011; Hillman et al., 2008]; note that such factors were not measured in this study). There were, however, no significant associations in the opposite direction: broad measures of brain size, either total volume or specific volumes of grey matter, white matter, or WMH, did not serve as an early indicator of impending cognitive decline, despite in some cases predicting more volume loss.

The neural mechanisms underlying our findings are unclear. There is not, for instance, a consensus on the explanation for the relation between WMH presence and growth and reduced cognitive ability; a recent review discusses possibilities ranging from WMHs damaging important neural networks, disrupting neurotransmitters such as those involved in the cholinergic system, or merely acting as an indicator of poorer vascular health, which may impair cognitive ability for other reasons [Prins and Scheltens, 2015]. Further insights into the cytoarchitecture of WMH and the accompanying changes in so‐called normal appearing white matter are provided in Muñoz Maniega et al. [2015] and in Wardlaw et al. [2015]. It is possible that multiple mechanisms operate and interact: it is unlikely that any one single mechanism is responsible for the loss of complex cognitive abilities. The precise mechanisms for the relation between loss of ‘healthy’ brain tissue (which, as the reviews above suggest, is not necessarily ‘healthy’) and cognitive decline are also unknown: since brain volume correlates with the number of neurons [Pakkenberg and Gundersen, 1997] as well as with the number of other key cells that are equally as essential, it may be that volumetric losses index decreases in the number of cells available to form the networks required for complex, distributed cognition, with function affected at multiple points.

### Comparison with Previous Studies

In a review of cross‐sectional samples, Walhovd et al. [2011] showed that over the entire lifespan, declines in the volume of grey matter were stronger than those for white matter. However, during the age range covered in the present study (the eighth decade of life), they found more white matter loss. Walhovd et al.'s estimates were expressed in terms of decades, so a direct comparison to the span of our study (73‐76 years) is not possible, but our findings—that grey matter declined by around 0.6% across the three years, and white matter by around 1%—are consistent with their reported pattern. WMH growth, which in our sample occurred at an expectedly faster rate than in previous analyses of younger individuals [e.g. Schmidt et al., 2005], was also associated with more atrophy of healthy grey and white matter. There was only a very small, non‐significant correlation (*r* = 0.060) between the rates of decline in grey and normal‐appearing white matter in our sample. This separable decline in grey and white matter raises the possibility that grey and white matter declines might provide differential information about cognitive ageing. The change‐change association between grey matter and WMH confirms and extends previous cross‐sectional work from this sample [Aribisala et al., 2013] and other cohorts [Tuladhar et al., 2015]. Tracking the separate declines across longer periods may reveal dissociable determinants and outcomes of these changes. Incidentally, Figure 3 illustrates that a minority of individuals showed apparent increases in their healthy brain tissue volumes (and decreases in WMH). For instance, 60 of the 459 individuals with repeated data showed increases in total brain volume (mean increase = 11.40 cm^3^, SD = 11.31). It is not possible to know whether these represent true volume increases or simply measurement error.

Our results for loss of healthy grey and white matter accord with those of a previous study by Kramer et al. [2007]. They showed, in a sample of 50 adults assessed across a near‐identical period of life to the participants in the present study (from approximate mean age 74 years to 78 years, though with a wider range than our sample), that grey matter atrophy was associated with declining executive function (a composite measure that included one of the same tasks used here to index Fluid Intelligence, WAIS Digit Span Backward). Also consistent with our results, Kramer et al. [2007] found no link between total grey matter volume decline and reductions in memory, indexed by tasks similar to ours, including a list‐learning test.

Our results are not consistent with some other studies of the brain volumetric substrates of cognitive ageing. The null result for coupled change in total brain volume and memory is contrary to the result of Schmidt et al. [2005] (*n* = 329), who showed that decline in memory, among other abilities, was linked to loss of total brain volume over six years (though in participants a decade younger on average than ours). Charlton et al. [2010] (*n* = 84) found no relation of total brain volume loss with two‐year change in any cognitive ability (although, perhaps unexpectedly, only working memory skills showed significant longitudinal decline in that study). The inconsistent results, in some cases the small sample sizes, and the heterogeneity of ages and cognitive tests included across previous studies make it difficult at present to obtain a broad picture of the pattern of brain‐cognitive associations in ageing. Further, large‐sample analyses, such as the present study, will lead to more solid conclusions.

The numerically (and sometimes significantly) strongest results in our study were found for change‐change correlations with cognitive abilities and WMH. In line with the conclusions drawn from the Kloppenborg et al. [2014] meta‐analysis, we found that WMH increase occurred alongside decline across all cognitive domains. However, the size of the effects we found were larger than their meta‐analytic change‐change average for Processing Speed (−0.33 compared to −0.10) and for Memory (−0.17 compared to −0.07), and were outside of their 95% confidence interval estimate in both cases. Our estimate for Fluid Intelligence (−0.25) was within the confidence intervals they calculated for General Intelligence (−0.50 to −0.02). As noted above, the widely‐varying estimates from previous studies come from the use of different tests, and in some cases single indicators, of cognitive ability. Previous work has also used a range of methods to quantify WMH. Our latent‐variable analysis in a narrow‐age cohort, capturing the change variance common across five highly valid and reliable processing speed tests, along with our standardized, validated measure of WMH, is likely to have produced more reliable results.

Whereas Fluid Intelligence and Speed changes had no significant differences in the sizes of their correlations with change across the three tissue types, Memory decline was more significantly related to WMH change than it was to healthy white matter atrophy. Indeed, in our sample, only WMH change was correlated with decline in memory. After excluding individuals with possible dementia, an interesting pattern emerged: the change‐change relations with grey matter and WMH remained significant, but those for normal‐appearing white matter were substantially reduced, and became non‐significant. This analysis suggests that relations between atrophy of white matter and cognitive ability might be of more relevance in individuals with dementia, rather than those experiencing normal cognitive decline.

We found a small number of significant sex differences: aside from male participants showing more decline in total brain and grey matter volumes than females, males showed stronger level‐change correlations between Speed and total brain volume, Speed and white matter volume, and Memory and white matter volume. Males also had a stronger change‐change correlation between Speed and white matter volume. The sex difference analyses in the present paper were, however, necessarily exploratory: although our unusually‐large sample allowed us to test these differences, we did not have a strong hypothesis that led us to expect differences in the size of the correlations in either direction. Researchers interested in differential brain and cognitive ageing by sex may wish to follow up on these analyses in large neuroimaging cohort studies.

A previous analysis of this sample used diffusion tensor MRI to index the brain's white matter microstructure (fractional anisotropy) across twelve white matter tracts [Ritchie et al., 2015]. Three‐year decline in fractional anisotropy in these 12 tracts was significantly related to decline in Fluid Intelligence, but not Processing Speed. Given the established connection between measures of speed and white matter structure [Bucur et al., 2008; Lövdén et al., 2014; Penke et al., 2012], we might have expected to find coupled changes in speed and diffusion tensor parameters, rather than macrostructural measures as used in the present study, but this was not the case for our sample. Comparing these two analyses emphasizes the fact that overall changes in macrostructural brain volumes provide different information about cognitive ageing than changes in microstructural diffusion characteristics in specific areas of the brain's white matter.

### Strengths and Limitations

The present study used data from a large sample, with modest attrition: just over 70% of the 657 participants who had brain volumetric data available at age 73 returned to provide follow‐up data at age 76. The time of life assessed—the eighth decade of life—is when risk for dementia increases substantially [Matthews and Brayne, 2005]: knowing more about the neuroanatomical underpinnings of cognitive decline during this period of life will allow better distinction between pathological and non‐pathological states of decline. As discussed above, the sample had a narrow age range, minimizing the confounding effects of age at each measurement [Hofer and Sliwinski, 2001]. They were assessed on a wide range of cognitive tests that produced latent cognitive change factors for each domain that were free of test‐specific measurement error. Within the model, we tested not just the strength of the associations, but the significance of the differences between the effect sizes. These factors all increase the comprehensiveness of our results.

Although the sample was large, it was not fully representative of the general population: typical selection biases meant that the participants were likely more intelligent and healthier on average than the general population of individuals in their 70s [Johnson et al., 2011]. All of the cognitive tests had substantial loadings on the latent factors we extracted (most loadings around *λ = 0*.5), and the measurement model was well‐fitting, but it is possible that different combinations of variables (for instance, only those variables which tap working memory, some of which we included in Fluid Intelligence and some of which were indicators of Memory) would produce somewhat different results. The Memory measures, which showed less decline than the other cognitive tests, may have been more subject to practice effects (the same story was used in the Logical Memory test at each wave, and the same word pairings in the Verbal Paired Associates test), which may have masked stronger memory decline. Whereas all the brain measures and cognitive factors showed significant change across the three years of the study, a longer follow‐up period would have allowed us greater power to detect coupled change [Rast and Hofer, 2014]. In addition, further data collection points will allow researchers to test for nonlinear trajectories of change in brain tissue volumes and cognitive abilities. We did not include an analysis of dropout: we used maximum likelihood modeling to allow us to use all the data, but if there were systematic (for example, medical) reasons for dropout, this may have biased our results. Indeed, the participants who did not return tended to have lower cognitive ability than those who returned (though their brains only showed significant differences in grey matter volume): they would thus be predicted to have had poorer general health [e.g. Deary et al., 2010]. For this reason, our follow‐up may have missed those with the greatest degree of brain atrophy, and underestimated the true effect sizes of the correlations.

## CONCLUSIONS AND FUTURE DIRECTIONS

Our comprehensive analysis of a longitudinal birth cohort shows that examining later‐life atrophy in gross measures like total brain volume or grey and white matter volumes is informative about cognitive decline, and confirms that WMH growth is a particularly useful indicator of cognitive change. The effect sizes for the level‐change and change‐change correlations were usually modest: the largest effect size was *r* = 0.334 for correlated change in WMH volume and Processing Speed. This is perhaps not surprising given the relatively short time period of the study.

Larger portions of the variance in cognitive ageing are likely to be explained by more complex patterns of differential change in specific brain regions of interest, such as the hippocampus [Apostolova et al., 2012; Papp et al. 2014], other areas of the medial temporal lobe [Murphy et al., 2010], the corpus callosum [Ryberg et al., 2011], and areas of the prefrontal lobes [Maillet and Rajah, 2013], the latter of which we might expect—along with parietal areas—to relate most strongly to tests of fluid intelligence, given the strong evidence for the Parieto‐Frontal Integration Theory of intelligence [Jung and Haier, 2007]. Examining WMH growth in specific areas of the brain, for instance using methods that reliably can tell apart deep and periventricular WMH, may also reveal more nuanced relations with cognitive change [Prins and Scheltens, 2015]. In addition, given the strong correlated change we observed amongst the cognitive abilities, it may be fruitful in future studies to examine an overall, general factor of cognitive change [e.g. Tucker‐Drob, 2011]—though it should be noted that latent factors of fluid intelligence often correlate very strongly with general intelligence [Kan et al., 2011]—and possibly to use bifactor models to find associations between neural variables and specific abilities that are unrelated to this general factor.

Combining the explanatory power of these volumetric measures with that from diffusion MRI measures [Madden et al., 2009; Nakagawa et al., 2013] as well as functional imaging [Grady, 2012] and imaging genetics [Wang et al., 2015] is a major task for future research into cognitive ageing. As Raz and Lindenberger [2011] have argued, the longitudinal approach taken in the present study is the most reliable way of uncovering these important relations between brain and cognitive decline.
